# Transformation of sodium bicarbonate and CO_2_ into sodium formate over NiPd nanoparticle catalyst

**DOI:** 10.3389/fchem.2013.00017

**Published:** 2013-09-18

**Authors:** Mengnan Wang, Jiaguang Zhang, Ning Yan

**Affiliations:** Department of Chemical and Biomolecular Engineering, National University of SingaporeSingapore

**Keywords:** hydrogenation, carbon dioxide, sodium bicarbonate, sodium formate, nickel, palladium, nanoparticles, heterogeneous

## Abstract

The present research systematically investigated, for the first time, the transformation of sodium bicarbonate and CO_2_ into sodium formate over a series of Ni based metal nanoparticles (NPs). Ni NPs and eight NiM (M stands for a second metal) NPs were prepared by a facile wet chemical process and then their catalytic performance were evaluated in sodium bicarbonate hydrogenation. Bimetallic NiPd NPs with a composition of 7:3 were found to be superior for this reaction, which are more active than both pure Ni and Pd NPs. Hot filtration experiment suggested the NPs to be the truly catalytic active species and kinetic analysis indicated the reaction mechanism to be different than most homogeneous catalysts. The enhanced activity of the bimetallic nanoparticles may be attributed to their smaller size and improved stability.

## Introduction

Utilizing renewable resources is a prerequisite for a sustainable society. One easily available renewable carbon resource is carbon dioxide (CO_2_), which has the advantages of being non-toxic, abundant, and economical (Sakakura et al., 2007). Unfortunately, few running industrial process employs CO_2_ as a building block as CO_2_ is the most oxidized state of carbon and therefore difficult to be activated. However, increasing efforts have been devoted to the transformation of CO_2_ into value added substances including methane (Riedel et al., 2003; Sai Prasad et al., 2008; Sharma et al., 2011), methanol (Imai et al., 1995; Kilo et al., 1997; Barrault and Urresta, 1999; Natesakhawat et al., 2012; Wesselbaum et al., 2012), formaldehyde (Lee et al., 2001), formic acid (Inoue et al., 1976; Yin et al., 2001; Elek et al., 2003; Himeda et al., 2003, 2006; Laurenczy et al., 2007; Tanaka et al., 2009; Federsel et al., 2010a,b; Sanz et al., 2010; Boddien et al., 2011a,b; Hao et al., 2011; Himeda et al., 2011; Peng et al., 2011; Preti et al., 2011; Wan-Hui and Yuichiro, 2012; Ziebart et al., 2012) and organic carbonates (Tomishige et al., 2001; Li and Zhong, 2002; La et al., 2007; Dai et al., 2009; Kohno and Sakakura, 2009; Lee et al., 2011; Ghazali-Esfahani et al., 2013). Among them, formic acid, conveniently obtained by hydrogenation of CO_2_, is highly attractive as formic acid is an important chemical used as preservative and antibacterial agent in food as well as the tanning of leather. Moreover, formic acid has been regarded as a clean source for H_2_ generation (Fellay et al., 2009; Boddien et al., 2011a; Himeda et al., 2011).

In aqueous solution, the hydrogenation of CO_2_ into formic acid is thermodynamically unfavorable. Hence many base additives such as KOH, NEt_3_ are used to promote this reaction. NaHCO_3_ is another starting material that could be used to overcome the thermodynamic limitation. Currently, the research activity on the reduction of CO_2_ into formic acid is predominantly focused on homogeneous catalysts. A variety of organometallic catalysts based on Ru (Yin et al., 2001; Elek et al., 2003; Himeda et al., 2006; Laurenczy et al., 2007; Federsel et al., 2010b; Hao et al., 2011; Himeda et al., 2011; Wesselbaum et al., 2012), Ir (Tanaka et al., 2009; Schmeier et al., 2011), Rh (Himeda et al., 2003, 2011), or Fe (Sai Prasad et al., 2008; Federsel et al., 2010a; Boddien et al., 2011b; Ziebart et al., 2012) have been developed for the hydrogenation of CO_2_. The classical Wilkinson catalyst (RhCl(PPh_3_)_3_) and the Ru analogue (RuCl(PPh_3_)_3_) were firstly introduced by Inoue et al. in 1976 (Inoue et al., 1976). These two complexes exhibited turnover number (TON) of 22 and 87, respectively, under room temperature and 50 bar H_2_/CO_2_ (1/1) in the presence of triethylamine for 20 h, which were more active than other catalysts being tested. Following this work, various Rh and Ru catalyst bearing phosphine and nitrogen ligands have been investigated. For example, Joó et al. reported the [RuCl_2_(tppms)_2_]_2_ (tppms = sodium diphenylphosphinobenzene-3-sulfonate) which exhibited a turnover frequency (TOF) of 54 h^−1^ at 50°C and 10 bar H_2_. The meritorious catalytic activity of Ir complexes has only been reported recently, although the initial study on Ir catalysts could date back to 1976 (Inoue et al., 1976). Tanaka and his colleagues reported a remarkable TOF of 150000 h^−1^ with PNP-Ir trihydride complex as the catalyst (Tanaka et al., 2009). Catalysts based on non-precious metals have also been explored. To date, the most active iron catalyst for the hydrogenation of carbon dioxide and bicarbonate was reported recently by Carolin et al. (Ziebart et al., 2012). Employing iron(II)-fluoro-tris[(2-(diphenylphosphino)phenyl)phosphino]tetrafluoroborate as the catalyst, the hydrogenation of bicarbonates results in good yields with high catalyst productivity and activity (TON >7500, TOF >750). Interestingly, Beller et al. reported a catalyst, [RuCl_2_(C_6_H_6_)]_2_/dppm (dppm = 1,2-bis(diphenylphosphino)methane), which is formed *in-situ* and can catalyze both the hydrogenation of bicarbonate and the dehydrogenation of formate in H_2_O/THF (Boddien et al., 2011a). As such, a hydrogen storage system based on the inter-conversion of formate and bicarbonate that is controlled by one catalyst becomes possible. There are several excellent reviews providing more comprehensive and detailed information for CO_2_ hydrogenation over homogeneous catalysts (Jessop et al., 1995; Scott et al., 2005; Himeda, 2007; Wan-Hui and Yuichiro, 2012).

Although homogeneous catalysts proved to be very successful in terms of achieving high catalytic activity, they are intrinsically associated with a few limitations. For instance, the ligand and the catalyst are sometimes not readily available and/or the costs are high. Removing the catalyst from product is also very difficult, which makes the recycle and reuse challenging. These limitations can be easily overcome by using heterogeneous catalysts. The very first approach to CO_2_ hydrogenation into formic acid was carried out by Farlow and Adkins in 1935 using Raney nickel as a catalyst under 20–40 MPa and at 353–423 K (Farlow and Adkins, 1935). Afterwards, very few heterogeneous systems have been reported. Preti et al. investigated CO_2_ hydrogenation in the presence of neat NEt_3_ to form HCOOH/NEt_3_ adducts over gold black (Preti et al., 2011). Tsang and co-workers explored the possibility of using copper zinc oxide catalyst for the methyl formate production from CO_2_ (Kerry Yu and Tsang, 2011). There were several investigations on the heterogenization of homogeneous catalysis by immobilizing ruthenium complex catalysts on silica and polystyrene resin (Baiker, 2000; Zhang et al., 2004). Besides, Peng et al. have conducted density functional theory (DFT) calculations on CO_2_ hydrogenation to formic acid on Ni(111) (Peng et al., 2011). Nevertheless, hydrogenation of carbon dioxide (or bicarbonate) into formic acid over heterogeneous catalyst has not been systematically attempted.

Nanoparticle (NP) catalysts represent an area where traditional homogeneous catalysis and heterogeneous catalysis converge (Yan et al., 2010a). It has been known for many years that the size of metal particles immobilized on solid supports in heterogeneous catalysts are often of nanoscale dimensions, nevertheless, recent advances in nanoscience enable the precise characterization of NPs and open the way for a further enhancement of their catalytic properties (Yuan et al., 2012). One of the main branches of nanocatalysis is catalysis by “soluble” NPs in liquid phase. NP catalysis in traditional solvents originated from colloidal chemistry but is now well-advanced as an independent discipline of research (Roucoux et al., 2002). Generally these dispersed NP catalyst exhibited increased activity under mild reaction conditions. A majority of previous studies were focused on nobel metal NPs and more attention should be paid to first-row transitional metals such as nickel, as these metals are more abundant and much more affordable. Herein, we report a series PVP stabilized Ni and NiM (M stands for the second metal) NPs in aqueous solution, which can effectively transform carbon dioxide and sodium bicarbonate to formic acid under mild condition.

## Materials and methods

### Materials

Sodium bicarbonate (99.99%) was purchased from AnalaR NORMAPUR; sodium formate (98.0%) was obtained from Fluka; sodium borohydride (95.0%) was from TCI; polyvinylpyrrolidone (PVP, M.W. = 40000) was from Alfa Aesar rhodium (III) chloride hydrate (Rh 38.5–45.5%) was from Alfa Aesar; Fe(II) chloride tetrahydrate (98%) was from Singma Aldrich; palladium(II) chloride (Pd 59.50%) was from Shaanxi Kaida Chemical Engineeering Co., Ltd.; auric chloride acid (Au 50%), chloroplatinic acid (Pt 47%), nickel(II) chloride hydrate (98.0%), copper(II) chloride (99%) and ruthenium(III) chloride hydrate (Ru 37%) were from Sinopharm Chemical Reagent (SCR). CO_2_ (99.998%) and H_2_ (99.995%) were purchased from was from Singapore Oxygen Air Liquide Pte Ltd. (SOXAL). All chemicals were used as received.

### Catalyst preparation

For preparation of Ni, Ni_8_Ru_2_, Ni_8_Rh_2_, Ni_8_Au_2_, Ni_8_Pt_2_, Ni_8_Fe_2_, Ni_8_Ag_2_, and Ni_8_Cu_2_ NPs, the desired amount of metal precursor (1.2 mmol, for bimetallic NPs, the Ni:M ratio was 4:1 with a total metal content of 1.2 mmol) and PVP (2.664 g, 20 molar equivalent) are dissolved in H_2_O (30 ml). NaBH_4_ (0.2269 g, 6 mmol) was dissolved in H_2_O (10 ml) and was added into the metal salt-PVP solution in one portion. The color of the solution turned to dark immediately indicating the reduction of the metal precursors. The final solution was transferred into a reactor (100 ml) when effervescence ceased.

For NPs containing Pd, PdCl_2_ (1.073 g, 6 mmol) was first dissolved in HCl solution (15 ml, 2 M) to obtain H_2_PdCl_4_. After that, the solution was dried using a rotary evaporator (IKA, RA10) to remove water and excess HCl. The remaining H_2_PdCl_4_ is dissolved in H_2_O (25 ml) to form a 0.24 M solution. For Ni_7_Pd_3_, Ni (II) chloride hexahydrate (0.200 g, 0.84 mmol) were dissolved together with H_2_PdCl_4_ solution (1.5 ml, 0.24 M) and PVP (2.664 g, 24 mmol) in H_2_O (30 ml). NaBH_4_ (0.2269 g, 6 mmol) were dissolved in H_2_O (10 ml) and added into the metal salt-PVP solution immediately. The color of the solution turned to dark immediately indicating the reduction of the metal precursors. The final solution was transferred into a reactor (100 ml) when effervescence ceased. Pd, Ni_8_Pd_2_, Ni_6_Pd_4_, Ni_4_Pd_6_, and Ni_2_Pd_8_ NPs were prepared in a similar way except the ratio of Ni and Pd was adjusted to the desired value.

### Catalyst characterization

Transmission electron microscopy (TEM) images were taken on a JEOL JEM-2010 microscope operating at 200 kV. TEM samples were prepared by diluting fresh prepared catalyst solution (0.05 ml) with methanol (1.5 ml), following which one drop of the solution was placed on a carbon film covered copper grid and dried under air.

X-ray photoelectron spectroscopy (XPS) measurements were performed on a VG ESCALAB MKII spectrometer, using mono Al Kα X-ray source (hν = 1486.71 eV, 5 mA, 15 kV) and calibrating N 1 s (N in PVP) to 399.70 eV. A thick PVP layer on the NP surface would prevent the photoelectrons from the metals to be detected. As such, NPs for XPS measurement were prepared with reduced PVP (5 instead of 20 equiv.). Fresh NP solution (4 ml) was mixed with acetone (50 ml). The mixture was centrifuged and viscous semi-solid material at the bottom of the centrifugation tube was collected, dried under vacuum, and analyzed by XPS.

X-Ray Powder Difraction (XRD) was performed on a Bruker D8 Advanced Diffractometer with Cu Kα radiation at 40 kV. The XRD sample was prepared by freeze drying the fresh NP solution (4 ml) as described in the catalyst preparation section.

### Hydrogenation of bicarbonate

Catalytic reactions were carried out in a high-pressure reactor (100 ml). NaHCO_3_ (1.008 g, 12 mmol) was added into the prepared nanoparticles solution. The reactor was pressurized with H_2_ (20 bar) and heated to 80°C for 2 h with a stirring speed of 250 rpm. After the reaction, the reactor was cooled down to room temperature and then the pressure was released. In some cases, CO_2_ (5–30 bar) was simultaneously purged into the reactor before the reaction.

### Product analysis

Product analysis was performed on an Agilent 1200 Series (Agilent Technologies, Germany) LC system equipped with a binary pump, an online degasser, an auto plate-sampler, and a thermostatically controlled column compartment. Chromatographic separation was carried out at 50°C on an Agilent ZorBax Hi-Plex H column (7.7 mm × 300 mm). The mobile phase used was H_2_SO_4_ aqueous solution (0.005 M). The flow rate was kept at 0.75 ml/min, run time was 15 min and the UV detector was set at 220 nm. The sample volume injection was 20 μL. A series of sodium formate was prepared (0.05 M, 0.10 M, 0.15 M, and 0.20 M, respectively). These standard solutions were used to generate a calibration curve with a *R*^2^ of 0.999.

## Results and discussion

**Figure 1:**
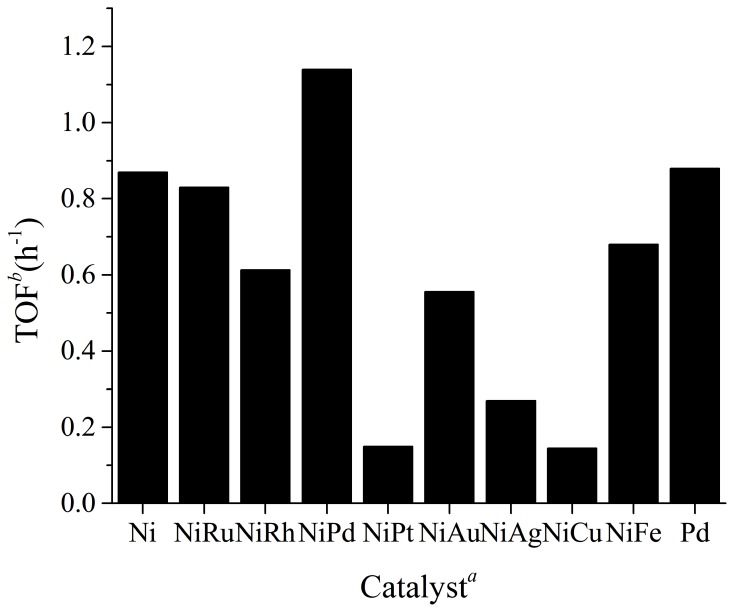
**TOF of Ni, Pd, and NiM NPs in the hydrogenation of sodium bicarbonate to sodium formate.** Reaction conditions: 0.012 mol NaHCO_3_, 1.2 mmol catalyst, 24 mmol PVP, 40 ml H_2_O, 20 bar H_2_, 80°C, 2h. ^a^All the NiM were prepared with the precursor molar ratio Ni: *M* = 4:1. ^b^TOF was defined as (the mole of sodium formate)/(the mole of metal · reaction time).

Ni is an ideal metal for the hydrogenation of CO_2_, as it is cheap, abundant, and in principle magnetically recoverable and recyclable. It is among the first catalysts that are known to be active for this reaction, however, there is still no systematic investigation concerning the catalytic activity of Ni containing NPs in CO_2_ hydrogenation. As such Ni and eight other NiM (M represents a second metal, and the mole ratio between Ni and M was fixed to be 4:1) NPs were prepared with a classical solvent reduction method by employing PVP as the stabilizer and NaBH_4_ as the reducing agent in water (Yan et al., 2009b, 2010b). Following that the catalytic activity of these NPs in the hydrogenation of sodium bicarbonate into sodium formate was evaluated in the presence of 20 bar hydrogen under 80°C (see Figure 1). Pure Ni NPs were active for the reaction, with a TOF of 0.87 being observed, which is significantly more active than that of Raney Ni catalysts (Farlow and Adkins, 1935). The superior catalytic activity of PVP stabilized Ni NPs is not unexpected, as metal catalysts with smaller particle sizes generally display improved catalytic performance due to the increased percentage of surface atoms. Interestingly, the activity of the catalysts dropped in most cases when the second metal is introduced. Pt, Ag, and Cu, in particular, exhibited very strong inhibition effect. On the other hand, NiPd NPs were more active than pure Ni NPs. As a control, we synthesized pure Pd NPs under identical conditions and found that they had a comparable activity with pure Ni NPs but were less active than bimetallic NiPd NPs. Hence, it is demonstrated that the NiPd bimetallic catalyst to be superior over the monometallic catalysts as well as bimetallic catalysts with other metal combinations.

**Figure 2:**
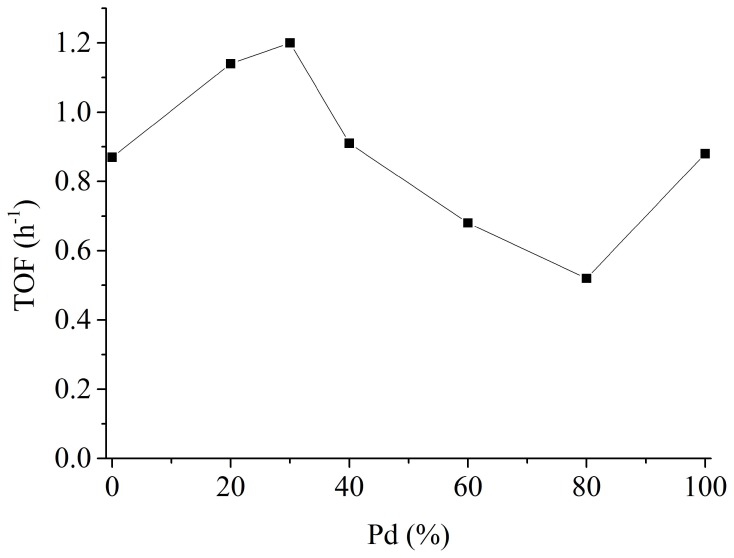
**TOF of NiPd NPs as a function of Pd content.** Reaction conditions: 0.012 mol NaHCO_3_, 1.2 mmol catalyst, 24 mmol PVP, 40 ml H_2_O, 20 bar H_2_, 80°C, 2h.

In the next step, we synthesized NiPd NPs with different metal ratio, simply achieved by modifying the amount of the two metal precursors before reduction. NiPd NPs with five different compositions were prepared and subjected to catalytic test. The results are compiled in Figure 2. For easy comparison the TOF of pure Ni NPs and Pd NPs are also plotted. A volcano type of relationship between the metal ratio and the catalytic activity was observed. NiPd NPs with a Pd content of 30% (denoted as Ni_7_Pd_3_ NPs) were most active (1.20 h^−1^). Although the TOF of NiPd NPs is not comparable to the most active homogenous complexes, it is one of the highest values for heterogeneous catalysts reported to date.

In some cases, metal NPs only act as the pre-catalysts for a reaction. A good example is the Pd NP catalyzed C-C coupling reaction in which the Pd(II) leached from the NP surface are believed to be the catalytic active species (Yin and Liebscher, 2006; Yan et al., 2009a). To confirm whether the reaction was truly catalyzed by heterogeneous Ni_7_Pd_3_ NPs a hot filtration experiment, initially developed by Maitlis et al. (Widegren and Finke, 2003) to distinguish homogeneous catalyst from heterogeneous ones, was carried out. The reaction was conducted as normal, quenched at 30 min, and analyzed by HPLC (45% sodium formate yield). Then the Ni_7_Pd_3_ NPs were removed by ultra-filtration and the remaining solution was used for catalysis under identical conditions. The yield of sodium formate remained unchanged (44.8%) after 1 h, indicating the reaction ceased after removal of Ni_7_Pd_3_ NPs. In addition, most homogeneous catalysts involve metal hydride as the key intermediate in the catalytic cycle. However, employing NMR to detect the presence of metal hydride in the solution was unsuccessful. These experiments provide strong evidences that the heterogeneous Ni_7_Pd_3_ NPs were responsible for the observed catalytic activity.

**Figure 3:**
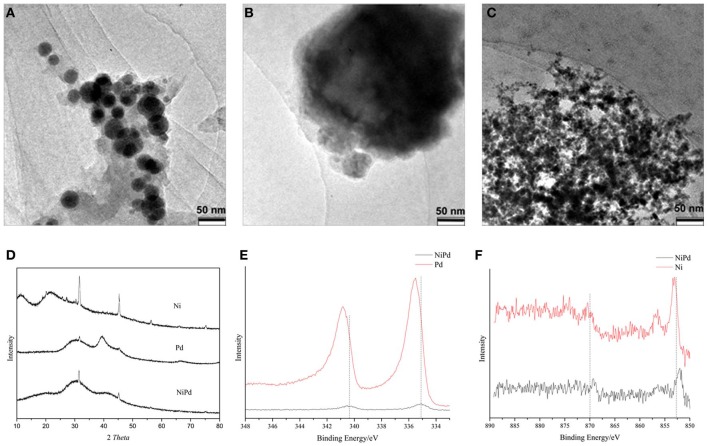
**TEM images of (A) Ni, (B) Pd, and (C) Ni_7_Pd_3_ catalysts. (D)** XRD patterns of Ni, Pd, and Ni_7_Pd_3_. **(E)** Pd 3d and **(F)** Ni 2p XPS spectra for Ni_7_Pd_3_, Ni, and Pd.

To rationalize the superior catalytic activity of NiPd NPs in sodium formate hydrogenation, TEM, XRD, and XPS analysis were conducted. Figures 3A–C are the representative TEM images of the Ni, Pd, and Ni_7_Pd_3_ NPs. These NPs were not well-separated from each other which makes the accurate determination of their sizes challenging. Nevertheless, it is apparent that pure Ni (20~40 nm) and Pd (~200 nm) NPs were significantly larger than Ni_7_Pd_3_ (<10 nm) NPs.

Figure 3D shows the XRD pattern of PVP stabilized Ni, Pd, and Ni_7_Pd_3_ NPs. For Ni NPs, the broad bands centered at around 2θ = 11 and 20° are characteristic of PVP polymer (Yuan et al., 2011). In the other two patterns, the broad peak at around 2θ = 30° is due to the presence of NaBO_2_—a byproduct in the preparation of catalyst. In all the three patterns, the peaks at 2θ = 31.7, 45.5, 56.5, 66.2 and 75.3° were observed and are due to the presence of NaCl. Pd metal exhibit XRD peaks at 2θ = 40.1 and 46.6° respectively, while for the Pd NPs the peaks slightly shifted to higher angle, in agreement with previous observation for PVP stabilized Pd NPs (Drelinkiewicz and Hasik, 2001). For both Ni and Ni_7_Pd_3_ NPs, no peak that can be ascribed to metal or metal oxide crystallite was observed, indicating they are amorphous materials, which is not uncommon for metal NPs reduced by NaBH_4_(Fan et al., 2009).

The XPS spectra of the Ni, Au, and Ni_7_Pd_3_ NPs are shown in Figures 3E,F (Ni 2p and Pd 3d regions). The binding energy (BE) of Pd 3d_5/2_ in Pd NPs was 335.5 eV, and it was 335.1 eV in Ni_7_Pd_3_ NPs. Both values are close to that of Pd(0) NPs prepared in other systems (335.3 eV), (Yuan et al., 2013) suggesting the Pd atoms are in the zero valent state. Nevertheless, the electron density in Ni_7_Pd_3_ NPs is slightly higher than that in pure Pd NPs. The BE of Ni 2p_3/2_ was 853.1 eV in Ni NPs and 852.0 eV in Ni_7_Pd_3_ NPs. Note that the BE of Ni 2p_3/2_ of Ni metal is 852.7 eV, the valent state of Ni in both NPs is metallic. Similar to Pd, the electron density of Ni in Ni_7_Pd_3_ NPs is higher than that in Ni NPs. Both Ni and Pd metal atoms are more electron-enriched in bimetallic NPs, which cannot be explained by mutual electron transfer between them, but instead indicating the Ni_7_Pd_3_ NPs are more stable against oxidation upon storage. Based on the peak intensity the ratio between Ni and Pd in the Ni_7_Pd_3_ NPs was calculated to be 3:5, dramatically lower than the stoichiometric ratio of 7:3. As XPS is a surface sensitive technique, it is plausible that the Ni_7_Pd_3_ NPs adopt a Pd enriched outer layer, core-shell structure. This structure can be rationalized by the fact that Pd has a lower surface energy and is in consistent with previous reports on the NiPd NPs. For example, Bertolini (Michel et al., 1998) and Creemers (Helfensteyn et al., 2003) discovered that the Pd surface composition of the PdNi alloy equilibrated at 600°C is higher than 80%. And the Ni_79_Pd_21_ and Ni_29_Pd_71_ NPs (with diameter 2–3 nm) exposed 53 and 91% of Pd atoms on the surface, respectively (Faudon et al., 1993; Renouprez et al., 1997).

It is widely accepted that the catalytic synergetic effect of two transitional metals in contact originates either from the so-called “ensemble effect” or the “ligand effect,” which describe the geometric and electronic modifications induced by the system constituents, respectively (Sinfelt et al., 1972). Ensemble requirement is known as the number of metal atoms required per adsorbed molecule for the most favorable path of a catalytic reaction (Sachtler, 2008). In bimetallic systems, the second metal introduced could effectively block certain ensembles but leave others intact, thus the catalytic activity would be changed due to the modifications of these ensemble. Alternatively, the second metal (termed as B) can be regarded as a “ligand” adjoining to the main surface atoms (termed as A). The bond between the surface atom A and its adsorbed molecules will be affected by the electronic interactions of B alloying with A, which is called ligand effect. NiPd bimetallic catalyst has been successfully employed in selective hydrogenation of buta-1,3-diene, (Massard et al., 2007) nitro-substituted aromatics, (Raja et al., 2005) CO_2_-methane dry reforming, (Steinhauer et al., 2009) formic acid electrooxidation (Li et al., 2011) and C-H bond cleavage reaction (Li et al., 2013). The enhanced catalytic performance of NiPd NPs were ascribed to the electron donation of Ni to Pd, (Ruban et al., 1997) the compressive strain of Pd atoms to adapt the Ni bulk lattice, (Filhol et al., 2002) and the unusual surface structure caused by surface relaxation (Filhol et al., 2001). In our system, however, these factors are unlikely to be the major reason for the superior activity of Ni_7_Pd_3_ NPs. XPS analysis indicates there is no clear evidence for the electron donation from Ni to Pd. XRD reveals the Ni_7_Pd_3_ NPs are amorphous, suggesting a majority of Pd and Ni atoms do not adapt a bulk lattice, and therefore should not suffer a significant compressive strain.

Based on TEM, XPS, and XRD analysis, we attribute the improved catalytic performance of Ni_7_Pd_3_ NPs to two main factors. First of all, the Ni_7_Pd_3_ NPs are smaller in size compared to pure Ni and Pd NPs, resulting in a higher exposer of surface atoms for catalysis. The second reason is that zero valent atoms on the NP surface are responsible for the catalytic activity. Ni_7_Pd_3_ NPs are more stable against oxidation so that they have a higher percentage of zero valent sites for the hydrogenation of sodium bicarbonate. It has been demonstrated previously that in the synthesis of bimetallic NPs from two metal salts, the presence of a noble metal salt could significantly promote the reduction of the 3-d metal such as Ni (Wang and Li, 2010). In our study, it appears that the incorporation of Pd not only promote the reduction of Ni salts during synthesis, but also stabilize the zero valent Ni atoms during storage and reaction. Unfortunately, we are not able to find a proper explanation for this effect and have to study in more detail in the future.

**Figure 4:**
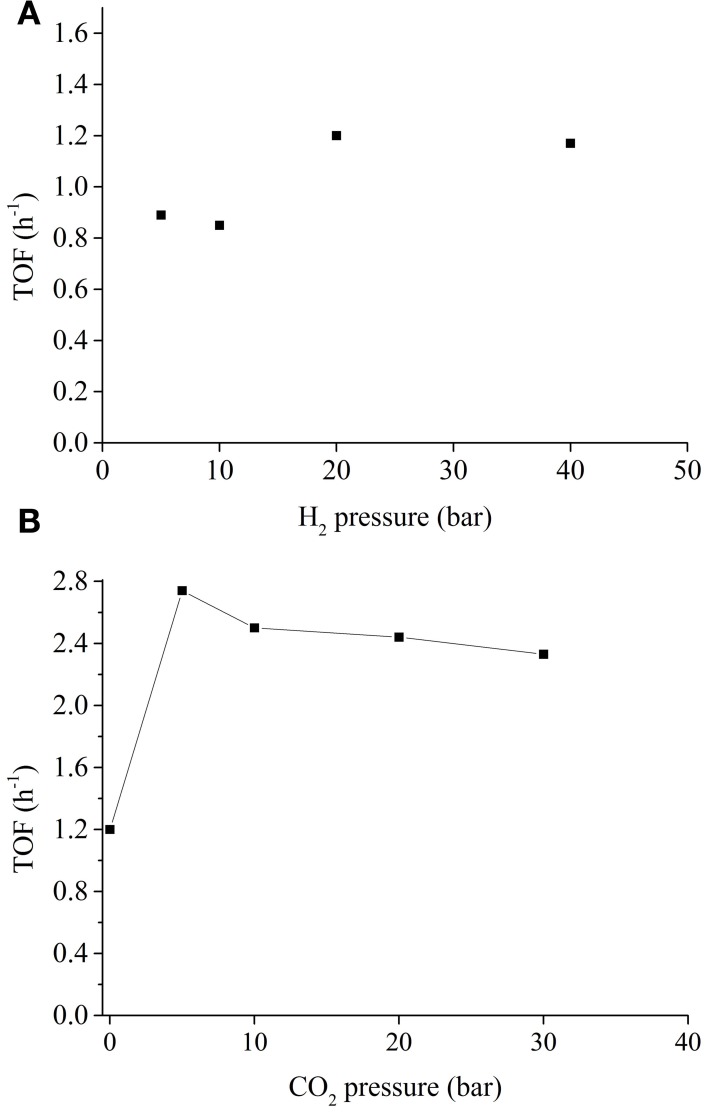
**(A)** TOF as a function of H_2_ pressure. Reaction condition: 0.012 mol NaHCO_3_, 1.2 mmol Ni_7_Pd_3_, 0.024 mol PVP, 40 ml H_2_O, 80°C, 2 h; **(B)** TOF as a function of CO_2_ pressure. Reaction condition: 0.012 mol NaHCO_3_, 1.2 mmol Ni_7_Pd_3_, 0.024 mol PVP, 40 ml H_2_O, 40 bar H_2_, 80°C, 2 h.

For CO_2_ hydrogenation into formic acid over homogeneous catalysts, the reaction rate is usually a function of CO_2_ and H_2_ gas pressure. In most cases, the reaction is of first order with respect to H_2_ pressure, (Tsai and Nicholas, 1992; Hutschka et al., 1997) although there is a report indicating at higher pressures (>60 bar) saturation kinetics was observed (lower than first order) (Thomas et al., 2001). We analyzed the catalytic activities of Ni_7_Pd_3_ NPs with varying H_2_ pressure (see Figure 4A). At the range of 5–40 bar, the TOF of the NPs were essentially irrelevant to the H_2_ concentration, suggesting the mechanism of the reaction over Ni_7_Pd_3_ NPs are fundamentally different from those over homogeneous catalysts. For homogeneous catalysts, the key step(s) in the entire catalytic cycle is generally the formation of the metal hydride, (Ziebart et al., 2012) which necessitates a high hydrogen pressure. Unlike the homogeneous catalysts, Ni (Yu and Hou, 2013) and Pd (Tungler and Fogassy, 2001) metals are well known for their ability to absorb and dissociate H_2_ molecules on the surface under very mild conditions. Thus, H_2_ activation is not the rate determine step and the concentration of H_2_ is not a major factor to influence the catalytic activity.

**Figure 5:**
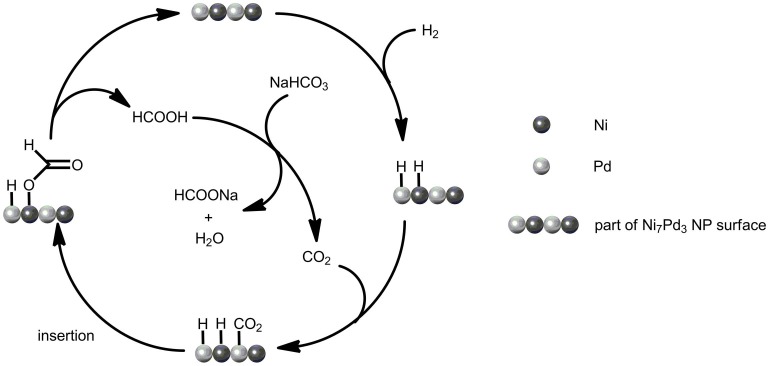
**Proposed reaction mechanism of CO_2_ hydrogenation into formate over Ni7Pd3 NPs**.

The relationship between catalytic activity and CO_2_ pressure was also explored (see Figure 4B). Introducing CO_2_ into the system has a significant promotion effect on the reaction rate. In the presence of 5 bar CO_2_, the TOF of the Ni_7_Pd_3_ NPs increased sharply to 2.8, as compared to 1.2 under CO_2_ free conditions. This is in agreement with previous reports using homogeneous catalysts where the system became more active when a mixture of CO_2_ and H_2_ gas was used instead of pure H_2_ (Himeda et al., 2004, 2007; Federsel et al., 2010b). A further increase in CO_2_ concentration has negligible effect on the reaction rate. The enhancement of TOF implies that the reaction mechanism might be modified in the presence of CO_2_. Probably, CO_2_ is more active than bicarbonate for the hydrogenation over Ni_7_Pd_3_ NPs. When CO_2_ is present in the system, it is CO_2_ instead of bicarbonate anion that inserts into the metal-hydrogen bond to form formate. The major function of sodium bicarbonate is to react with the formic acid generated to make the transformation thermodynamically favorable (see Figure 5).

## Conclusion

For the first time, transformation of sodium bicarbonate and CO_2_ into sodium formate over eight Ni based metal NPs were systematically investigated. NiPd NPs with a Ni:Pd ratio of 7:3 was identified to be the most active catalyst for this reaction. Under low temperature and base free conditions, the optimized TOF of the Ni_7_Pd_3_ NPs in sodium bicarbonate hydrogenation reaches 2.8 h^−1^, which is among the highest for heterogeneous catalysts. The superior catalytic activity of the Ni_7_Pd_3_ NPs over pure Ni and Pd NPs were ascribed to the smaller size of the bimetallic NPs and the stabilization effect of Pd to Ni atoms. Kinetic study indicates that H_2_ is easily activated over the Ni_7_Pd_3_ NPs which is fundamentally different from homogeneous catalysts. Although many homogeneous complexes with much higher catalytic activity are known, the robustness of this system, the use of inexpensive metals and the heterogeneous nature (which means facile recycling) make the Ni_7_Pd_3_ NPs attractive, and worth further investigation, in CO_2_ hydrogenation.

### Conflict of interest statement

The authors declare that the research was conducted in the absence of any commercial or financial relationships that could be construed as a potential conflict of interest.
